# Radiosynthesis of 6’-Deoxy-6’[^18^F]Fluorosucrose via Automated Synthesis and Its Utility to Study *In Vivo* Sucrose Transport in Maize (*Zea mays*) Leaves

**DOI:** 10.1371/journal.pone.0128989

**Published:** 2015-05-29

**Authors:** David Rotsch, Tom Brossard, Saadia Bihmidine, Weijiang Ying, Vikram Gaddam, Michael Harmata, J. David Robertson, Michael Swyers, Silvia S. Jurisson, David M. Braun

**Affiliations:** 1 Department of Chemistry, University of Missouri, Columbia, Missouri, United States of America; 2 Division of Biological Sciences, Interdisciplinary Plant Group and the Missouri Maize Center, University of Missouri, Columbia, Missouri, United States of America; 3 University of Missouri Research Reactor, University of Missouri, Columbia, Missouri, United States of America; Leibniz-Institute for Vegetable and Ornamental Crops, GERMANY

## Abstract

Sugars produced from photosynthesis in leaves are transported through the phloem tissues within veins and delivered to non-photosynthetic organs, such as roots, stems, flowers, and seeds, to support their growth and/or storage of carbohydrates. However, because the phloem is located internally within the veins, it is difficult to access and to study the dynamics of sugar transport. Radioactive tracers have been extensively used to study vascular transport in plants and have provided great insights into transport dynamics. To better study sucrose partitioning *in vivo*, a novel radioactive analog of sucrose was synthesized through a completely chemical synthesis route by substituting fluorine-18 (half-life 110 min) at the 6’ position to generate 6’-deoxy-6’[^18^F]fluorosucrose (^18^FS). This radiotracer was then used to compare sucrose transport between wild-type maize plants and mutant plants lacking the *Sucrose transporter1* (*Sut1*) gene, which has been shown to function in sucrose phloem loading. Our results demonstrate that ^18^FS is transported *in vivo*, with the wild-type plants showing a greater rate of transport down the leaf blade than the *sut1* mutant plants. A similar transport pattern was also observed for universally labeled [U-^14^C]sucrose ([U-^14^C]suc). Our findings support the proposed sucrose phloem loading function of the *Sut1* gene in maize, and additionally demonstrate that the ^18^FS analog is a valuable, new tool that offers imaging advantages over [U-^14^C]suc for studying phloem transport in plants.

## Introduction

Carbon assimilated via photosynthesis in source leaves is transported to non-photosynthetic sink tissues through the phloem tissues of veins [1–4]. This process, also known as carbohydrate partitioning, is highly dynamic and is fundamental to plant growth and development. Carbohydrate partitioning is not only controlled by the metabolic needs of the plant, but is also affected by abiotic (e.g., drought, heat, salinity) and biotic (e.g., insect, bacteria, fungi) stresses [5–12]. Thus, it is crucial to be able to study carbohydrate dynamics *in vivo* under various environmental, genetic, or developmental conditions, and the use of radiotracers represents an attractive, non-invasive mechanism to achieve this goal.

Radiotracers have been used in plant science for more than 60 years, and there is a growing body of work and interest in using *in vivo* radiotracer imaging studies to trace sugar dynamics in plants [13–19]. A number of radioactive isotopes, both long-lived (e.g., ^14^C) and short-lived (e.g., ^11^C), have been used to study sugar transport and distribution in plants [15,20–24]. While the long half-life of ^14^C (t_1/2_ = 5.73x10^3^ yrs) makes it well suited for longer term experiments lasting days, weeks, months, or years, it is not possible to sequentially label and resolve the signal in the same plant multiple times. Moreover, because low energy beta particles from ^14^C-decay escape from thin plant tissues and will likely be absorbed by thicker tissues, its use for *in vivo* imaging work is not ideal. This is also the reason why ^14^C-radiolabeled plants are often destructively sampled and processed for quantitation, such as by liquid scintillation counting, which makes this isotopic analysis technique informative to study carbon allocation, but not appropriate to look at short-term transport dynamics in an intact plant [22,25,26]. Contrary to ^14^C, ^11^C has a short half-life of t_1/2_ = 20.33 min, which permits subsequent, repeated labeling of the same plant, but it also limits its use to short-term experiments [14,15,17,20]. Even though the short half-life makes ^11^C fit for *in vivo* studies of carbohydrate dynamics, ^11^C has several disadvantages, including 1) the requirement to produce it at or near the experimental site because of the short half-life, 2) the safety concerns resulting from its high energy gamma emissions, and 3) its relatively expensive cost compared to other isotopes [24,27,28]. Other short-lived radioactive isotopes have been used in plant research such as oxygen-15 (^15^O) with t_1/2_ = 2.03 min, nitrogen-13 (^13^N) with t_1/2_ = 9.96 min, and fluorine-18 (^18^F) with t_1/2_ = 109.8 min [27,29–31]. The latter, with its extended half-life, makes it more feasible than the other short-lived isotopes for dynamic imaging studies.

Fluorinated sugar derivatives offer opportunities for application in many areas of biology. One of the most widely used is 2-deoxy-2-[^18^F]fluoro-D-glucose, which is commonly used in medical studies for imaging cancer; however, few studies utilizing it have been reported in plants [32–34]. Since sucrose is the principle end product of photosynthesis and the carbohydrate transported through the phloem to heterotrophic tissues in many plants, using fluorinated sucrose derivatives, rather than hexoses, will be more biologically relevant to study photoassimilate dynamics in plants. The fluorinated derivatives of sucrose that have been reported include 3-deoxy-3-fluorosucrose [35], 4-deoxy-4-fluorosucrose [36], 6-deoxy-6-fluorosucrose [37], 1’-deoxy-1’-fluorosucrose [38,39], 4’-deoxy-4’-fluorosucrose [40], 236’-deoxy-6’-fluorosucrose [40], and 6,6’-dideoxy-6,6’-difluorosucrose [38,41]. The syntheses of these fluorosucrose derivatives are quite complex and several require chemoenzymatic methods involving glucose isomerase and sucrose synthetase. The enzymatic approach was recently applied to obtain the first ^18^F fluorinated derivative, 1’-[^18^F]fluoro-1’-deoxysucrose, in 26% yield [39]. We have recently reported the first purely synthetic approaches for the syntheses of 6’-deoxy-6’-fluorosucrose, 1’-deoxy-1’-fluorosucrose, and 6-deoxy-6-fluorosucrose [42–44]. Here, we report the first radiolabeling of sucrose with ^18^F at the 6’ position, and the utility of 6’-deoxy-6’[^18^F]fluorosucrose (^18^FS) in studying sucrose phloem transport in maize (*Zea mays*) leaves and specifically with sucrose transporter (SUT) proteins.

SUTs are membrane proteins, some of which localize to the plasma membrane of companion cells and/or sieve elements within the phloem, and catalyze the H^+^-coupled uptake of sucrose for long-distance transport [1,6,45–50]. SUTs have been classified into different types/groups based on their protein sequence and affinity for sucrose [1,45,48–52]. Group 1 SUT proteins contain high-affinity transporters, some of which are proposed to function as the major transporters that load sucrose into the phloem of monocot plants, such as maize, while Group 2 SUT proteins are unique to dicots (i.e., potato and Arabidopsis plants). In many dicot plants, genetic and biochemical evidence have established that SUT1-type proteins function in phloem loading [53–57]. However, the role of SUT1 proteins in monocots is less clear. For example, orthologs of rice (*Oryza sativa*) and sugarcane (*Saccharum* spp. hybrid) SUT1 do not appear to function in phloem loading of sucrose [58–60]. Although the maize SUT1 protein is 82% and 95% identical at the amino acid level to the rice and sugarcane SUT1 proteins, respectively, the maize SUT1 protein has biochemical activity and sucrose affinity consistent with a role in sucrose phloem loading [61,62]. Additionally, the biological function of SUT1 in maize was demonstrated when a *sut*1 mutant was isolated [22,63]. Compared to the wild-type plants, *sut1* mutants exhibited a severe reduction in plant growth and fitness, and also displayed the hyperaccumulation of carbohydrates in the leaves. However, rarely, the *sut1* mutant plants produced tassels and viable pollen, indicating that some sucrose transport from source leaves was achieved to sustain development. As shown by experiments with [U-^14^C]suc, *sut1* mutant plants displayed a greatly reduced transport ability compared to wild-type plants [22].

In this report, our objective was to develop a ^18^F-fluorinated analog of sucrose, and to determine whether ^18^FS can be used as a tracer in plant imaging studies to monitor sugar dynamics by comparing its transport in maize *sut1* mutants to wild-type plants. Fluorine-18 offers a tremendous advantage over ^14^C in that the sucrose transport can be observed in real-time under a variety of physiological conditions and genetic manipulations by positron emission tomography (PET) imaging [64]. In addition, 6’-fluorosucrose is a very poor substrate of the sucrose cleaving enzyme invertase, unlike [U-^14^C]suc; hence, ^18^FS will not readily undergo catabolism and be incorporated into carbohydrate metabolism [40,65].

## Materials and Methods

### Materials

[^18^F]Fluoride was produced by the (p,n) nuclear reaction on [^18^O]water (Rotem, Hyox 18 enriched water, ≥98% O-18, ≥99.9%) with 16 MeV protons using a GE PET Trace cyclotron. All other reagents and common solvents were obtained from Sigma Aldrich. Solvents used for water-sensitive reactions (i.e., CH_3_CN) were anhydrous grade and were stored in Sure/Seal bottles prior to use. The labeling precursor, 6’-*O*-trifluoromethanesulfonyl-2,3,4,6,1’,3’,4’-hepta-*O*-benzoylsucrose was prepared according to [42] and stored under an argon atmosphere and over DrieRite prior to use. Cryptand 222 (cryptofix, K222, etc.) was purchased from Huayi Isotopes (99%) and stored in amber vials with teflon-coated stoppers under an argon atmosphere prior to use. [U-^14^C]suc was obtained from American Radiolabeled Chemicals, St. Louis, MO, and stored at 4°C until use.

### Automated radiosynthesis of ^18^FS

**Fig 1:**
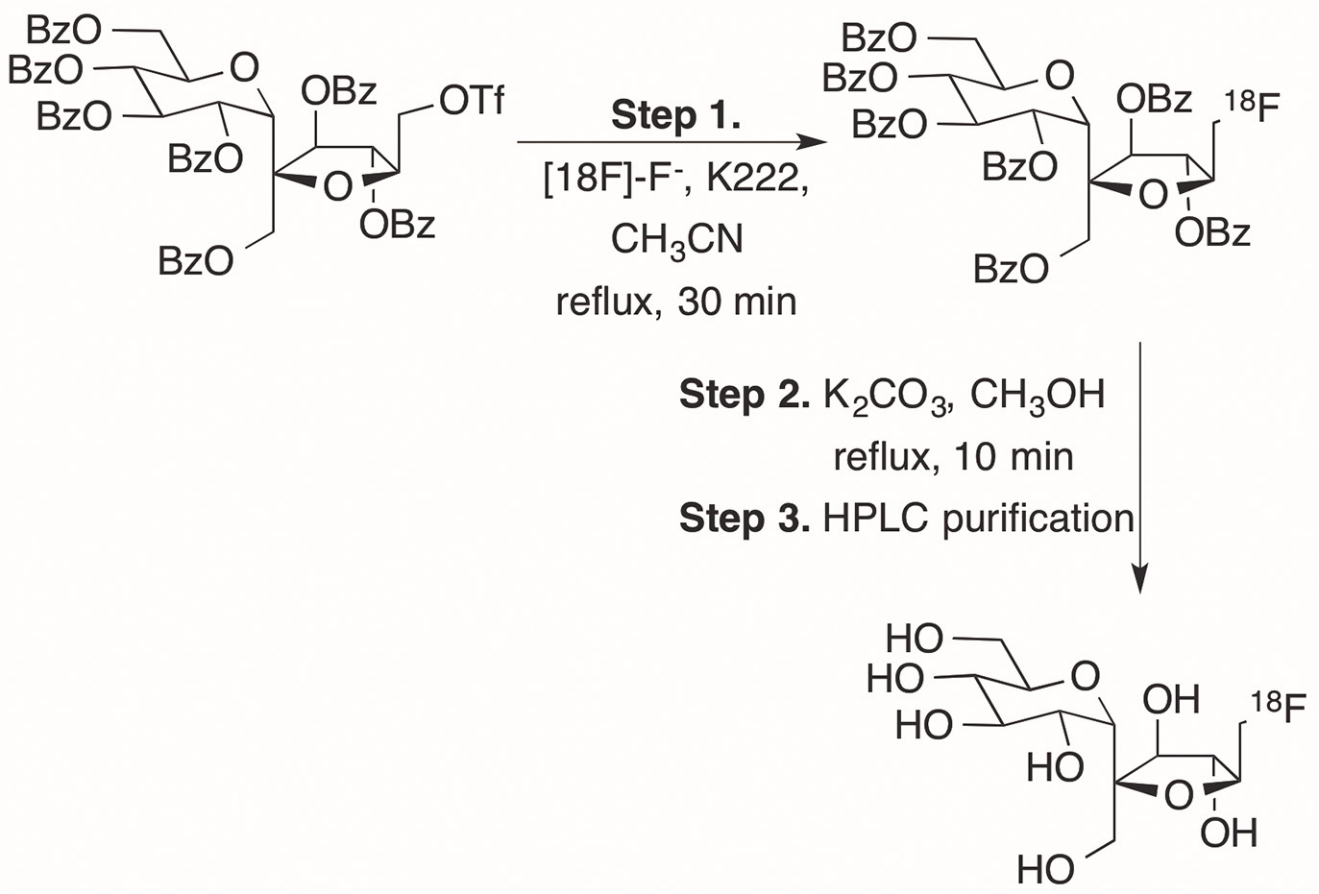
Synthesis of ^18^FS from a protected precursor.

The automated synthesis was performed on a dual reactor automated synthesis Modular-Lab system (Eckert & Zielger, Germany) as shown in Fig 1 and S1 Fig. All reagents and solvents were placed in the appropriate reservoirs under Ar_(g)_ prior to receiving [^18^F]fluoride from the cyclotron. Reservoir 1 was filled with K222 (15 mg in 1.0 mL CH_3_CN) and K_2_CO_3_ solution (2 mg/mL, 0.5 mL). Reservoir 2 was loaded with 6’-*O*-trifluoromethanesulfonyl-2,3,4,6,1’,3’,4’-hepta-*O*-benzoylsucrose (10–15 mg) dissolved in 1 mL of anhydrous CH_3_CN. Reservoirs 3 and 4 were filled with 1 mL of anhydrous CH_3_CN. Reservoirs 5 and 6 were empty and used for the purpose of adding Ar_(g)_ as a mixing gas. Reservoirs 7 and 8 were filled with K_2_CO_3_ in MeOH (5 mg/mL 0.5 mL) and HPLC mobile phase (75% CH_3_CN in water, 1 mL), respectively. Radioactivity in [^18^O]-water was received directly from the cyclotron through V1 into a receiving vial (marked as F-18/H_2_O in S1 Fig) using positive pressure (He_(g)_) from the cyclotron. Valve 1 (V1) was closed off to the system, the activity was then transferred using vacuum from the receiving vial to the ion exchange resin (QMA cartridge) through V2 and V3 to trap the [^18^F]fluoride. The [^18^O]-water was collected for recycling in a separate vial. The [^18^F] radioactivity was eluted from the QMA cartridge with K222/K_2_CO_3_ solution from the reservoir 1 into reactor 1 (R1) through V3 using vacuum. Water and solvent were evaporated from the radioactive fluoride by heating at 115°C in combination with Ar_(g)_ flow (100 mL/min) through V17 and V16 and vacuum through V8 and V7 for 10 min. The residual water was removed by azeotropic evaporation at 115°C, twice, for three min with anhydrous CH_3_CN transferred from reservoirs 3 and 4 through V5 (via Ar_(g)_ pressure), under vacuum and Ar_(g)_ flow (100 mL/min). The sugar triflate precursor dissolved in anhydrous CH_3_CN was added to the dried residue from reservoir 2 through V5 to R1 (via Ar_(g)_ pressure) and heated at 90^°^C for 30 min to fluorinate the precursor. The solution was cooled to 40°C and passed through an alumina neutral cartridge placed between R1 and R2. Once in R2, the solution was evaporated at 90°C under negative pressure and with Ar_(g)_ (100 mL/min) for 15 min. K_2_CO_3_ in MeOH was added from reservoir 8 to R2 through V10 (via negative pressure) and heated at 90°C for 10 min. Solvent was evaporated at 90^°^C under vacuum and Ar_(g)_ flow (100 mL/min). One milliliter of HPLC mobile phase (75% CH_3_CN, 25% water) was then added from reservoir 7 to R1 through V10 (via negative pressure).

### HPLC purification

**Fig 2:**
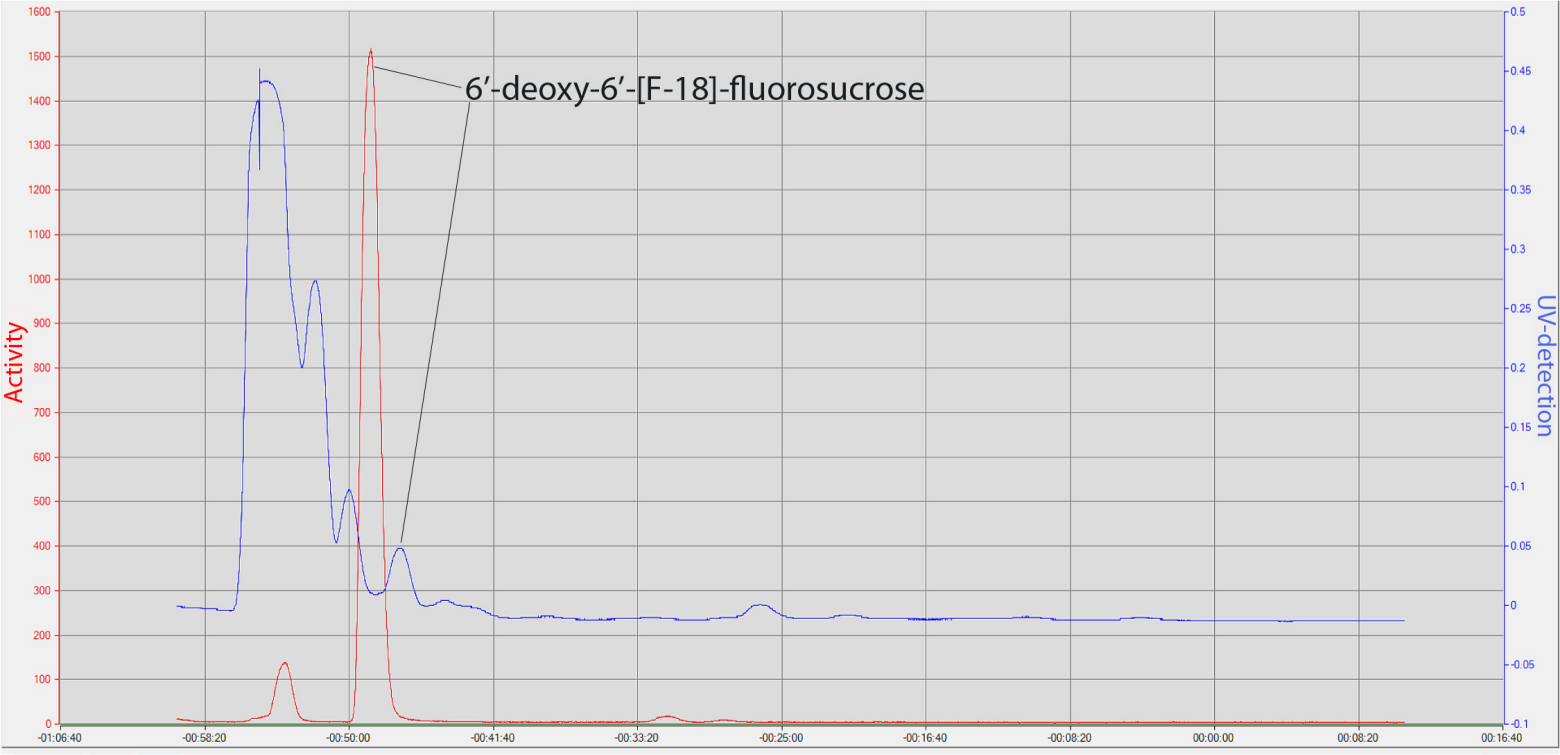
HPLC chromatogram of purified ^18^FS. Red line: radio-trace; Blue line: UV (193 nm) trace. The offset in time between the radio-peak and the UV trace is the time it takes the sample to pass through the loop in the activity detector and travel to the UV detector. Note time along the x-axis counts down. The sample was injected onto the HPLC at -60:00 min, and the ^**18**^FS peak on the radio-trace occurs around -49:00 min.

The automated system was programmed to allow a manual loop load and column injection. An exterior syringe was connected to the waste port of V12. The HPLC injection loop was manually loaded from R2 through V11 and V12 by applying negative pressure to the system using the attached syringe. A 4.6 x 250 mm^2^ with 5 μm particle size Ultra amino HPLC column (Restek, Bellefonte, PA, USA) was used for purification. The column was eluted with 75% CH_3_CN in 18.2 MΩ water at 2 mL/min. Fig 2 shows a representative HPLC chromatogram with both radiation and UV detection. The product was collected through V13 and V14 in the flask marked “Collection Flask HPLC Product.” The acetonitrile was evaporated from the product by heating at 85°C with Ar_(g)_ flow and vacuum through V14 and V13 (100 mL/min) for 10 min. Radioactivity of the final product was measured in a calibrated Capintec, CRC-25R radioisotope dose calibrator.

### Synthesis of ^18^FS for macroscopic analysis

The same procedure described above was followed to synthesize ^18^FS for macroscopic analysis except that 0.5 mg potassium fluoride (KF) was added to reservoir 1 prior to the start of the program. The reaction mixture was purified as described in the previous section. The purified product was collected, allowed to decay, and analyzed by NMR (^1^H, ^13^C, ^19^F) and ESI-MS.

### Plant growth conditions

Maize plants were grown in a greenhouse under ambient lighting. Plants were grown in pots in a peat-based potting soil (Promix BX, Hummert International, St. Louis, MO.), watered daily, and fertilized twice weekly with Peters 20:20:20 fertilizer. At a minimum of two days prior to the labeling, the plants were taken from the greenhouse and placed in a laboratory suitable for radiochemical studies to acclimate the plants to the new environment. The plants were placed under a halogen (120W) and two incandescent (65W) floodlights (335 μmols m^-2^ sec^-1^, measured at leaf level), maintained 12 hour day/ 12 hour night, and watered every other day. No fertilizer was added once the plants were removed from the greenhouse.

### Plant phenotyping and genotyping

The *sut1-m1* (abbreviated *sut1*) mutant allele was described previously [22]. Families segregating for *sut1* homozygous mutant individuals backcrossed into the maize inbred line B73 at least five times were used for all tracer studies. The *sut1* mutant plants were visually identified by their leaf chlorosis and anthocyanin accumulation. Genomic DNA was isolated, and mutant and wild-type plants were genotyped with a polymerase chain reaction (PCR) polymorphic primer set IDP8570F: 5’-CGCTAAGCTCGTCCTTCTCC-3’ and IDP8570R: 5’-GGTTTGTGATCTTTGTGTCACC-3’, which distinguished the mutant and wild-type alleles and is tightly linked to the *sut1* locus (located less than 1 Megabase away). The PCR reaction was performed by heating the DNA to 95°C for 2 min, followed by 35 cycles of 95°C for 30 sec, 60°C for 30 sec and 72°C for 1 min, and finally an elongation step at 72°C for 5 min. PCR products were resolved on a 2% agarose gel stained with SYBR Safe DNA Gel Stain (Invitrogen) and photographed on a Bio-Rad ChemiDoc XRS+ Gel Imaging Station. Genotyped homozygous mutant and wild-type plants were used for the study.

### ^18^FS transport assays

The second or third youngest expanded leaf, counting down from the top of the plant, on four-to-five week old plants was cut 2.5 cm from the tip of the leaf. The tip of the cut leaf was then dipped into a solution of radiolabeled sucrose (5 mM unlabeled sucrose, containing 150–200 μCi ^18^FS, 2 mL total volume) for 3 min. The leaf tip was then removed from the solution, and excess solution was wiped off the tip using a dry Kimwipe followed by a wet Kimwipe. The plant was allowed to sit for one hour under ambient conditions to perform photosynthesis as a chase to the ^18^FS pulse. Attempts to replicate the abrasion experiments previously reported using [U-^14^C]suc were difficult to consistently reproduce from plant to plant [22]. This approach gave irregular results and was abandoned in favor of the more consistent “cut and dip” method. After one hour, the labeled leaves were excised 22–25 cm from the tip, taped to a flat board with the abaxial surface of the leaf up, and the leaf was covered with Saran wrap. A phosphor plate (Fujifilm imaging plate BAS-MS 2025) was placed over the excised leaves and exposed for one hour. The experiment was repeated on four dates over the period of one month comparing seven wild-type and seven mutant plants using ^18^FS; separately, four sets of plants also received [U-^14^C]suc (see below). The phosphor imaging plates were scanned on a GE Typhoon FLA 9000 fast laser scanner using the Control Software (Version 1.0) under the phosphor image setting with the laser set to 635 nm and pixel size at 100 μm. The images were analyzed using the GE ImageQuant TL Toolbox program (Version 7.0). Statistical analyses were performed using SAS (version 9.3, Cary, NC, USA; SAS Institute, Inc. 1998). The analysis of variance via the mixed-model procedure (PROC MIXED, SAS) was used to determine significant differences in ^18^FS and [U-^14^C]suc transport between wild-type and *sut1* mutant plants. The plant type was considered fixed while the sampled plants within each type were considered random effects. Results are reported as mean ± standard error and significant differences between wild-type and *sut1* mutant plants were assessed at p < 0.05.

### ^18^FS and [U-^14^C]suc transport assays

A 2 mL, 5 mM unlabeled sucrose solution was spiked with 150–200 μCi ^18^FS and 125 μCi of [U-^14^C]suc for comparative transport studies. The selected leaf on an intact plant was treated as described above. After one hour of chase, the labeled leaves were excised, taped to a board with their abaxial surface face up, covered with Saran wrap, and imaged for one hour to detect the predominantly ^18^F signal. After twenty-four hours, all of the ^18^F had decayed, and another phosphor plate was placed on the leaves and left undisturbed for five days to image the [U-^14^C]suc and then analyzed as above.

## Results and Discussion

### Radiosynthesis of ^18^FS

**Fig 3:**
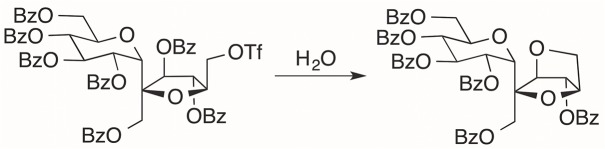
Internal conversion product of protected precursor.

Fig 1 represents the chemical synthesis scheme for ^18^FS. To our knowledge, this report is the first fully automated, non-enzymatic approach for the radiofluorination of sucrose. The precursor was developed and labeled with cold fluorine as previously described [42]. Translation of this chemistry to automated radiosynthesis afforded a decay corrected yield ranging from 1.8 to 23.6% of ^18^FS. The anhydrous [^18^F]F-K222 needed in step 1 of the synthesis took 41 min to generate while the synthetic time of step 1 was 30 min. Hydrolysis, transfer, and dry-down of the sample was completed in 22 min (step 2). HPLC separation, step 3, was completed in 12 min, and product dry-down and reformulation took 10 min. The total production time of ^18^FS averaged 115 min. The identity of the product was verified macroscopically by synthesis of the 6’-fluorosucrose with KF and a spike of ^18^F. The product was isolated from the reaction mixture by following the radio-peak associated with the ^18^F. The radio-HPLC peak was collected, allowed to decay, and analyzed by NMR (^1^H, ^13^C, ^19^F) and ESI-MS. A secondary product was observed in the UV spectrum of the radiolabeled peak collected in these experiments. Macroscopic analysis supports the side-product to be an intramolecular cyclization product from hydrolyzed starting material (Fig 3). The ratio of the cyclization product to 6’-fluorosucrose was one to two. The secondary product was not observed in the UV spectra when the synthesis was performed without KF.

Typical of S_N_2 reactions, this reaction was extremely water sensitive. Yields plummeted from an average of 10.6% to <1% when the precursor was not properly stored and the automated system was not properly prepared prior to synthesis. Proper preparation of the automated system includes rinsing the lines and reactor vessels with anhydrous acetonitrile and heating to dryness. Storing the precursor in single use vials sealed under argon gas also significantly increased yields. No differences in yields were observed whether using K222/K_2_CO_3_ or tetraethylammonium bicarbonate (TEAB) in the synthesis. Literature yields for cold labeling of sucrose were reported as >90% [42]. A common observation when translating cold labeling to radiolabeling is a significant decrease in yield [66–75]. Our product followed this trend with an average decay corrected radiochemical yield of 10.6%. In addition, the majority of automated syntheses incur chronically lower yields from inherent difficulties with automated systems [69,71,76–78]. Automation of radiosyntheses has advantages over manual syntheses such as lower radioactivity exposure and better reproducibility. However, automation suffers from several challenges, primarily the loss of activity and/or material during transfer steps and the inability to make changes during the synthesis. Our synthesis was not immune to transfer issues. Our activity levels were tracked throughout the synthesis by calibrated activity sensors within the system. We experienced losses as large as 20% of our initial activity at times, and these losses could occur at multiple steps before the HPLC purification. These losses account for our wide range of radiochemical yields and the lower yields compared to cold synthesis. One of the main losses in activity/material occurred during evaporation steps; white solid adhered to the walls of the reaction vessel at levels higher than the solvent reached for the following step, and thus, no reaction occurred with this solid. A simple remedy to this problem was to add more solvent to each consecutive step following evaporation. However, this resulted in more dilute reaction conditions and required longer future evaporations and did not increase yields. Manual syntheses allow for shaking or mixing by hand, which would dissolve any solid on the vessel walls. Addition of a stir bar to the automated system did not provide adequate agitation to wash reagents that had solidified on the vessel walls, and actually reduced yields by increasing spattering and bumping during evaporation steps.

It was found that increasing fluorination times from 10 min to 30 min improved yields. No significant change in yields was observed when the reaction temperature was raised from 90°C to 95°C. Deprotection of the fluorinated complex was not always complete in 5 min so the hydrolysis time was increased to 10 min. As indicated in the literature, fluorination yields may be dependent on different fluoride sources [42]. It is well-documented that sucrose undergoes hydrolysis to glucose and fructose under acidic conditions and that the rate of hydrolysis increases with temperature [79,80]. No hydrolysis products were observed in this work as the elution time of the radiolabeled peak was always consistent with that of sucrose (11 min) (Fig 2). Elution times for glucose and fructose under these conditions are 7 min and 4 min, respectively.

### ^18^FS transport is diminished in *sut1* mutant leaves

**Fig 4:**
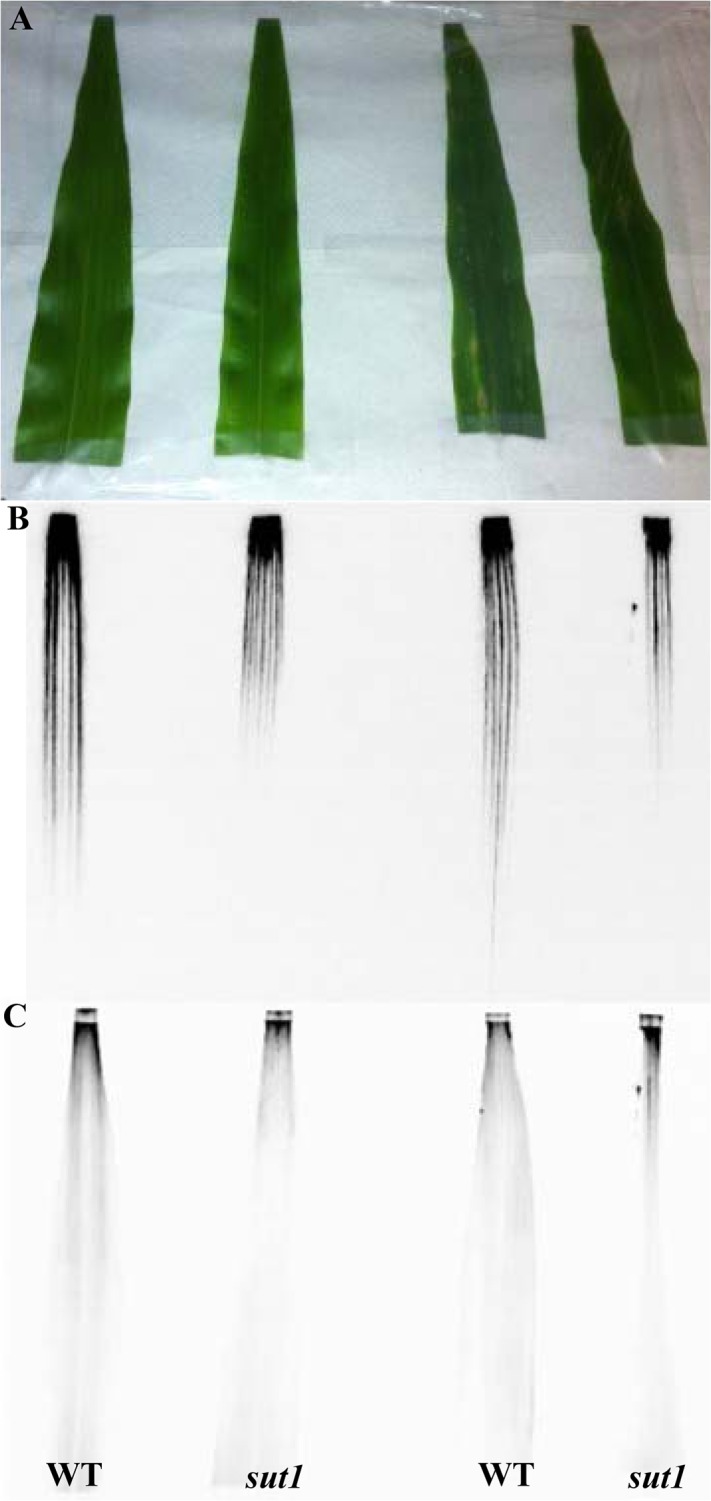
Transport of ^18^FS and [U-^14^C]suc in wild-type and *sut1* mutant leaves. A: Photo of leaves collected from two wild-type (WT) and two *sut1* mutant plants. B: ^**18**^F phosphor image of leaves exposed for one hour to a solution of 200 μCi ^**18**^FS and 125 μCi [^**14**^C]suc. C: ^**14**^C phosphor image obtained after five days exposure.

To determine whether ^18^FS would be transported by plants and suitable for *in vivo* imaging studies, we applied it to maize leaves. For these assays, recently matured source leaves of wild-type and *sut1* mutant plants were selected since these leaves were phenotypically similar to one another, and mutant leaves at this stage have similar carbohydrate levels and gas exchange properties to the wild type [22,63]. We deliberately did not use older, more mature leaves since *sut1* mutant leaves at this stage hyperaccumulate sugars and starch, display leaf chlorosis and anthocyanin accumulation, and are physiologically not comparable to the wild-type leaves [22,63]. A cut tip of a leaf of an intact plant was dipped into a solution of ^18^FS and then allowed to transport the tracer for one hour. The leaf was then excised and directly autoradiographed (Fig 4). The s*ut1* mutant plants exhibited substantially diminished transport of ^18^FS compared to wild type, as previously observed for [U-^14^C]suc [22]. One possible explanation for the reduced but detectable level of ^18^FS transport through the veins of the *sut1* mutant leaf blade is genetic redundancy. Six additional *Sut* genes are present in the maize genome, and another maize SUT protein may provide limited sucrose phloem loading activity in the absence of SUT1 function. Wild-type leaves showed considerably greater ^18^FS transport down the length of the blade towards the stem during the one hour transport time (Fig 4B).

**Fig 5:**
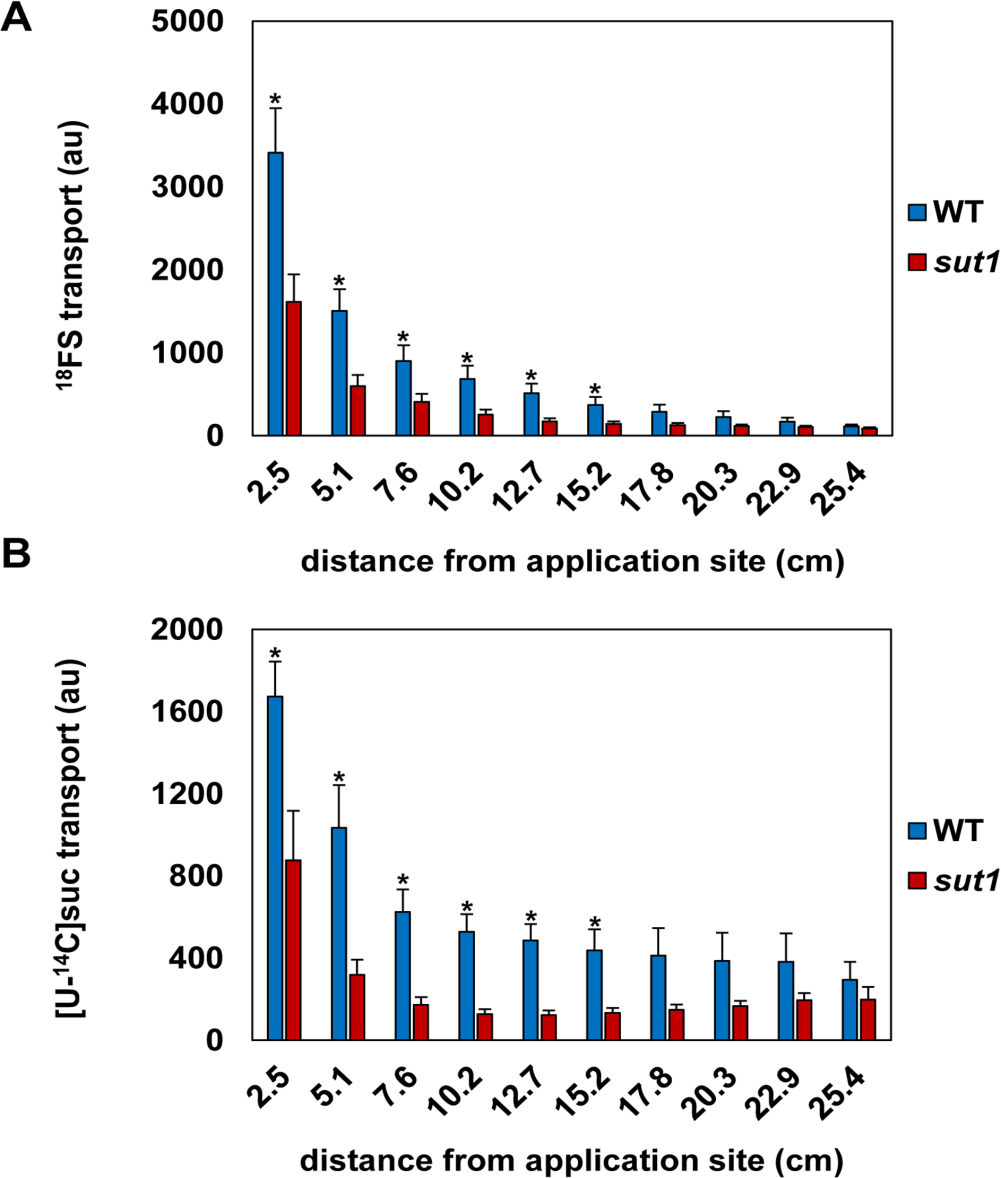
Quantification of ^18^FS and [U-^14^C]suc transport in wild-type and *sut1* mutant leaves. **A:** ^**18**^FS transport in *sut1* mutant and wild-type (WT) maize plants measured at different distances (in cm) from the application site. The graph represents an average transport intensity (in arbitrary units, au) ± the standard error at each distance. **B:** The graph represents an average transport intensity (in au) ± the standard error for [U-^**14**^C]suc for WT and *sut1* mutant plants. All leaves used in the [U-^**14**^C]suc imaging studies were also imaged using ^**18**^FS to compare their transport. An asterisk indicates that the value is significantly different from WT at p < 0.05.

Altering functional groups of substrates can greatly affect enzyme or transporter recognition [65,81–85]. Previous work has shown that 6’-deoxy-6’-fluorosucrose and 1’-deoxy-1’-fluorosucrose compete for binding at the same site as sucrose in a soybean (*Glycine max*) sucrose transport protein, and that both bind with approximately two-fold higher binding affinity than sucrose [81]. Furthermore, 1’-deoxy-1’-fluorosucrose is transported *in vivo* in soybean and maize [82]; however, even though it is bound at the same site and with similar affinity as 1’-deoxy-1’-fluorosucrose, to our knowledge, no data are available on whether 6’-deoxy-6’-fluorosucrose is transported by a SUT protein. To test whether the 6’-fluorinated sucrose analog behaves as a functional substrate of the maize SUT1 protein *in vivo*, universally labeled [U-^14^C]suc was spiked into a solution of ^18^FS, and the transport of this solution by the plant was observed. Both the ^18^FS and [U-^14^C]suc showed similar transport profiles (Fig 4C), with the *sut1* mutants demonstrating significantly less transport of either substrate down the length of the leaf blade than the wild type (Fig 5). The most parsimonious explanation for these data is that the Group 1 maize SUT1 protein is able to bind and transport 6’-fluorinated sucrose, similar to the Group 2 soybean SUT [40,65,81]. Further, this result supports the previous findings that the 6’ hydroxyl group of the fructose ring in sucrose is not essential for substrate binding or the transport mechanism [40,65,81]. However, although 6’-deoxy-6’-fluorosucrose is known to bind to SUT proteins and we observed a significant difference in ^18^FS transport between the wild-type and *sut1* mutant leaves, we cannot conclusively exclude the possibility that the ^18^FS is entering the phloem via some other indirect mechanism, which is not catalyzed by the SUT1 protein but nonetheless is impacted by the *sut1* mutation. Future *in vitro* studies testing SUT1 uptake kinetics for ^18^FS would be needed to distinguish between these possibilities.

**Fig 6:**
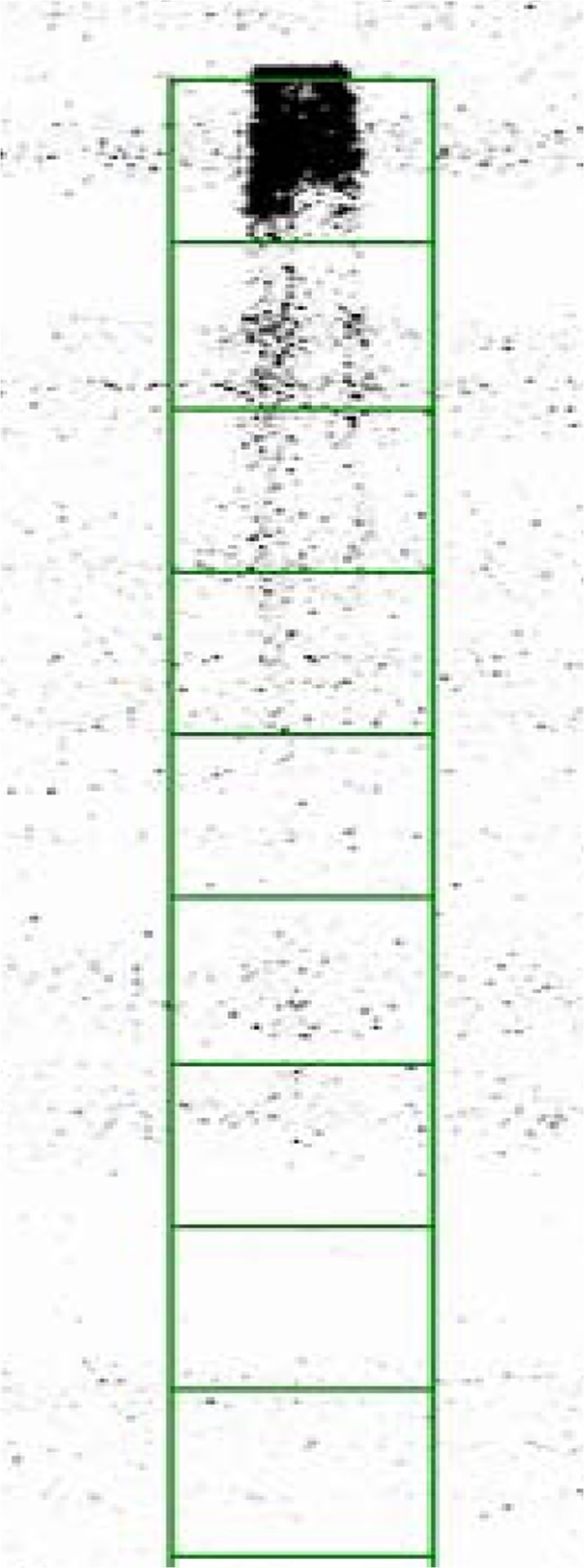
Transport of ^18^F as fluoride in wild-type leaves. 150 μCi of free ^**18**^F was applied to wild-type leaves and allowed to transport for two hours before a one hour image was obtained. Levels close to background were observed in the leaves with very low levels of transport.

The autoradiography data were quantified using the GE ImageQuant TL Toolbox program (Version 7.0). The amount of activity transported from the tip towards the stem was measured at different positions down the leaf (Fig 5). Fig 5A shows the transport mean of ^18^FS for the *sut1* mutant and wild-type plants. On average, the s*ut1* mutant leaves showed at least an approximately 53% decrease in ^18^FS transport from the application site to more than 15 cm down the leaf relative to the wild-type leaves (Fig 5A). Thus, these data suggest that the *sut1* mutant exhibited significantly reduced transport compared to the wild type. A similar decrease in transport down the leaf was observed in the [U-^14^C]suc measurements (Fig 5B). The lower lighting levels experienced by plants during the radiotracer transport assays likely accounts for the reduced transport differences compared with plants grown in higher light [53,55,86]. A radiograph of a wild-type leaf exposed to 150 μCi of ^18^F as fluoride in 5 mM sucrose shows no transport of ^18^F (Fig 6), demonstrating that the transport observed in these studies is labeled fluorosucrose and not free ^18^F ion.

While the transport patterns observed in the ^18^FS and [U-^14^C]suc images were similar in these experiments we did observe some differences. The images from ^18^FS display well defined vein loading and transport down the leaves toward the stem primarily through the veins (Fig 4B). However, the corresponding [U-^14^C]suc images show similar transport with minimal vein definition of the transport path (Fig 4C). There are several possible reasons why the signal from the ^18^FS labeled wild-type leaves appeared stronger and had more basal transport than the corresponding [U-^14^C]suc-labeled leaves. Some of the explanations are technical, such as we applied 200 μCi of ^18^FS versus only 125 μCi of [U-^14^C]suc to the leaves, so that the greater amount of radioactivity increased the relative ^18^FS signal. Alternatively, because ^18^F is a positron/gamma emitter, while ^14^C is a weak beta emitter, the ^18^F decay yielded a stronger signal that was easier to detect. However, an intriguing biological reason for the discrepancy may be that the ^18^FS is more poorly effluxed from the phloem than [U-^14^C]suc, and hence, less of the labeled sucrose is lost along the transport path. If so, this result might suggest that SWEET sucrose effluxers [5,87,88] are not as able to utilize ^18^FS as a substrate. Future work will be necessary to test this hypothesis.

Another difference we noted between the ^18^FS and [U-^14^C]suc signal in the wild-type leaves was that the [U-^14^C]suc activity was diffused and more wide-spread to the edges of the leaves. We hypothesize this could be due to lateral diffusion and/or metabolism of the [U-^14^C]suc throughout the leaves from application time to the time the [U-^14^C]suc image was completed five days later. This diffusion was absent from the ^18^FS images since the images are completed within only a few hours of application. The potential movement of the substrate post-harvest emphasizes the utility of imaging the ^18^FS tracer shortly after application in comparison with the longer time required to image the [U-^14^C]suc. Additionally, the ^18^FS images provide crisper details over the [U-^14^C]suc images.

## Conclusions

There is a growing interest in imaging sugar dynamics in plants using non-invasive radiotracers. We report a fully automated, two-pot, two-step synthesis of ^18^FS that has been achieved with reasonable yields and high purity. As observed with [U-^14^C]suc, plants lacking *Sut1* function displayed a significantly reduced export of ^18^FS from the leaves. This reduction was verified with a side-by-side comparison with [U-^14^C]suc. This report indicates the usefulness of ^18^FS as a tracer for sucrose transport through the phloem in maize leaves. Furthermore, it demonstrates that ^18^FS provides for shorter duration experiments and superior imaging signal compared with [U-^14^C]suc, making ^18^FS a useful, additional reagent for studying sucrose partitioning *in vivo*. Additionally, the data show that sucrose fluorinated on the 6’ position on the fructose moiety was similarly transported as [U-^14^C]suc, suggesting that the fluorine substitution at this position is tolerated by the group 1 maize SUT1 protein. The application of ^18^FS will greatly enable visualization of sucrose phloem transport dynamics in other carbon accumulating maize mutants [89–92], other plants, and under various environmental conditions, and it will allow for real-time imaging studies to be carried out using PET imaging systems [70,93].

## Supporting Information

S1 Fig Schematic diagram of the modular system for remotely controlled radiosynthesis of ^18^FS. A dual reactor automated synthesis Modular-Lab system (Eckert & Zielger, Germany) was used for the synthesis. The module used for the synthesis of ^18^FS is composed of eight different reagent reservoirs, two reactor vessels and an HPLC system. In this two reactor system, reactor 1 (R1) was used for the fluorination of the precursor and R2 was used for the deprotection step and reconstitution of the product in the HPLC mobile phase. Valves 1–3 controlled the capture and elution of [^18^F]fluoride into R1. The other reagents and solvents were controlled by V5 and V6 for R1 and V10 for R2. R1 was connected to reagent reservoirs, inert gas, and vacuum. R2 was connected to R1 through V6 and V9, reagent reservoirs, the HPLC valve, inert gas, and vacuum. Transfers out of R1 or R2 were performed using pneumatic lifts that were connected to V6 and V11. The lifts were in the up position during all reaction steps and reagent addition steps. The lifts were in the down position only when transferring out of the reactor vessels. Reservoir 1 was connected to R1 through V2 and V3, reservoirs 2–4 were connected to R1 through a 4-way valve, V5, and reservoirs 5 and 6 were connected to R1 through a 4-way valve, V6. Reservoirs 2–6 were pressurized by argon gas. Reservoirs 7 and 8 were connected to R2 through V10 and were open to atmosphere. R2 was connected to the HPLC valve (V12) through V11. The final product was collected after HPLC purification through V13 and V14. All other valves control appropriate operations as designed and necessary, such as transferring reagents or solvents and gas flow for evaporation purposes. The program and interface provides the user with control during appropriate steps of the synthesis (i.e., [^18^F]fluoride transfer from cyclotron to system, HPLC loading and injection, and collecting HPLC purified product). All transfer steps performed during the automated synthesis were achieved through positive argon gas pressure controlled by the flow control module or negative pressure produced from a diaphragm vacuum pump. Purification of the radiolabeled product was done by HPLC using an Ultra amino HPLC column (Restek, 4.6 x 250 mm^2^ with 5.0 μm particle size) with a pump integrated into the synthesis module, a Knauer UV-Detector Smartline UV-2500 operated at 193 nm, and an integrated activity detector ADII (Eckert & Ziegler, Germany). A solution of 75% CH_3_CN in H_2_O with a flow rate of 2.0 mL/min was used as the mobile phase for the HPLC purification of ^18^FS. All processes other than loading the HPLC were remotely controlled. Radioactivity of the final product was measured in a calibrated Capintec, CRC-25R radioisotope dose calibrator.
(TIF)
