# Supplementary figures and images for: Radiosynthesis of 6’-Deoxy-6’[18F]Fluorosucrose via Automated Synthesis and Its Utility to Study In Vivo Sucrose Transport in Maize (Zea mays) Leaves
Source: PLoS One. 2015 May 29;10(5):e0128989. doi: 10.1371/journal.pone.0128989 (PMC4449027; doi:10.1371/journal.pone.0128989)

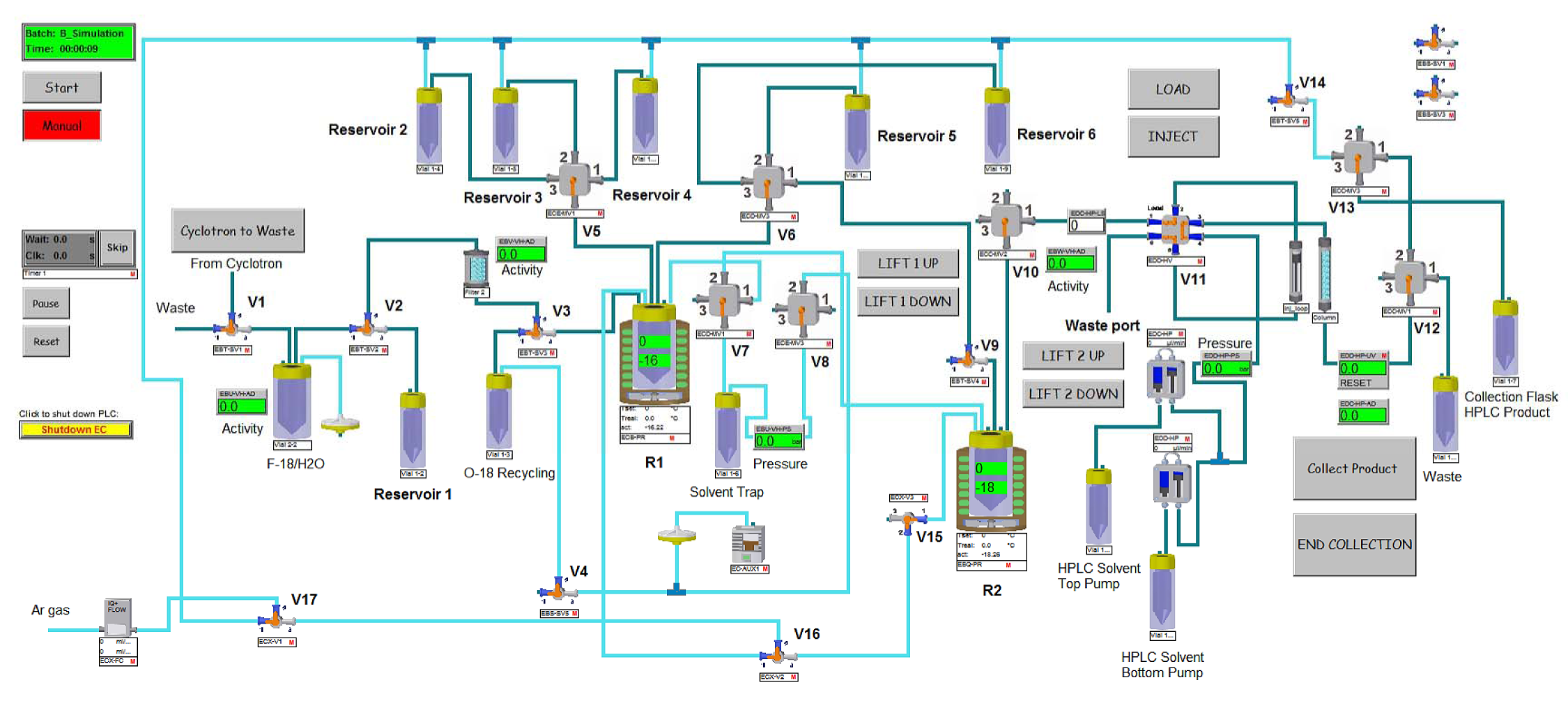

Supplement: S1 Fig — A dual reactor automated synthesis Modular-Lab system (Eckert & Zielger, Germany) was used for the synthesis. The module used for the synthesis of 18FS is composed of eight different reagent reservoirs, two reactor vessels and an HPLC system. In this two reactor system, reactor 1 (R1) was used for the fluorination of the precursor and R2 was used for the deprotection step and reconstitution of the product in the HPLC mobile phase. Valves 1–3 controlled the capture and elution of [18F]fluoride into R1. The other reagents and solvents were controlled by V5 and V6 for R1 and V10 for R2. R1 was connected to reagent reservoirs, inert gas, and vacuum. R2 was connected to R1 through V6 and V9, reagent reservoirs, the HPLC valve, inert gas, and vacuum. Transfers out of R1 or R2 were performed using pneumatic lifts that were connected to V6 and V11. The lifts were in the up position during all reaction steps and reagent addition steps. The lifts were in the down position only when transferring out of the reactor vessels. Reservoir 1 was connected to R1 through V2 and V3, reservoirs 2–4 were connected to R1 through a 4-way valve, V5, and reservoirs 5 and 6 were connected to R1 through a 4-way valve, V6. Reservoirs 2–6 were pressurized by argon gas. Reservoirs 7 and 8 were connected to R2 through V10 and were open to atmosphere. R2 was connected to the HPLC valve (V12) through V11. The final product was collected after HPLC purification through V13 and V14. All other valves control appropriate operations as designed and necessary, such as transferring reagents or solvents and gas flow for evaporation purposes. The program and interface provides the user with control during appropriate steps of the synthesis (i.e., [18F]fluoride transfer from cyclotron to system, HPLC loading and injection, and collecting HPLC purified product). All transfer steps performed during the automated synthesis were achieved through positive argon gas pressure controlled by the flow control module or [file pone.0128989.s001.tif]
